# Crystal Design, Antitumor Activity and Molecular Docking of Novel Palladium(II) and Gold(III) Complexes with a Thiosemicarbazone Ligand

**DOI:** 10.3390/ijms241411442

**Published:** 2023-07-14

**Authors:** Carolane M. Almeida, Érica C. M. Nascimento, João B. L. Martins, Tales H. A. da Mota, Diêgo M. de Oliveira, Claudia C. Gatto

**Affiliations:** 1 University of Brasilia, Institute of Chemistry, Laboratory of Inorganic Synthesis and Crystallography, Brasília 70904-970, Brazil; carolane.macedo@gmail.com; 2 University of Brasilia, Institute of Chemistry, Laboratory of Computational Chemistry, Brasília 70904-970, Brazil; ericamoreno@unb.br (É.C.M.N.); lopes@unb.br (J.B.L.M.); 3 University of Brasilia, Faculty UnB Ceilândia, Multidisciplinary Laboratory of Human Health, Brasília 72220-275, Brazil; talescara@gmail.com (T.H.A.d.M.); diegomadureira22@yahoo.com.br (D.M.d.O.)

**Keywords:** Pd(II) and Au(III) complexes, thiosemicarbazone, crystal structure, Hirshfeld surface, antitumor activity and molecular docking

## Abstract

The current research describes the synthesis and characterization of 2-acetylpyridine N(4)-cyclohexyl-thiosemicarbazone ligand (HL) and their two metal complexes, [Au(L)Cl][AuCl_2_] **(1)** and [Pd(L)Cl]·DMF **(2)**. The molecular structures of the compounds were determined by physicochemical and spectroscopic methods. Single crystal X-ray diffraction was employed in the structural elucidation of the new complexes. The complexes showed a square planar geometry to the metal center Au(III) and Pd(II), coordinated with a thiosemicarbazone molecule by the *NNS*-donor system and a chloride ion. Complex **(1)** also shows the [AuCl_2_]^−^ counter-ion in the asymmetric unit, and complex **(2)** has one DMF solvent molecule. These molecules play a key role in the formation of supramolecular structures due to different interactions. Noncovalent interactions were investigated through the 3D Hirshfeld surface by the *d_norm_* function and the 2D fingerprint plots. The biological activity of the compounds was evaluated in vitro against the human glioma U251 cells. The cytotoxicity results revealed great antitumor activity in complex **(1)** compared with complex **(2)** and the free ligand. Molecular docking simulations were used to predict interactions and properties with selected proteins and DNA of the synthesized compounds.

## 1. Introduction

Metal complexes have been extensively investigated after the success of the use of cisplatin as a chemotherapy treatment for several types of cancer, particularly those metal complexes that are isoelectronic and isostructural to platinum(II) complexes [1,2,3]. However, despite the similarity of the compounds, it is clear that it is impossible to indicate a specific behavior for this kind of metal complex, as each metal plays a different role in the body, and their properties depend on the type and coordination of the ligand present in the structure [4]. Gold compounds in +3 or +1 oxidation states have shown numerous pharmacological applications, such as antitumor [5,6,7], antimalarial [8] and antiparasitic [9]. Furthermore, works in the literature reported the great stability of Pd(II) complexes coordinated with sulfur and nitrogen atoms and showed that palladium compounds with thiosemicarbazones are potentially attractive as anticancer agents [10,11,12]. Both gold and palladium complexes have been demonstrated to be interesting in the study of the treatment of gliomas [13,14]. Therefore, the biological potential of metals can be directly linked to the high pharmacological capacity of their ligands [15].

Thiosemicarbazones are a class of ligands known for a long time to present many biological properties in their free form or coordinated with different metal ions. Many studies have reported that these ligands exhibit significant antibacterial and antitumor activity in vitro and in vivo, and are of great interest in developing therapeutic compounds, especially in cancer treatment, that act directly in the brain and other tissues [16,17,18,19]. Thiosemicarbazones also have the great advantage of their high versatility, as they possess both soft sulfur and hard nitrogen donor atoms that allow complexation with various metal ions in different geometries, including gold and palladium [20,21,22,23].

In this context, due to our interest in new metal complexes, we search to combine the pharmacological properties of thiosemicarbazone ligands with the metal centers Au(III) and Pd(II) [24,25]. The present work reports the study of the synthesis, spectroscopic and structural characterization of new metal complexes. All compounds were investigated by FTIR, UV–Vis and ^1^H NMR techniques. The complexes were analyzed by single-crystal X-ray diffraction and Hirshfeld surface analysis. Furthermore, molecular docking and cytotoxicity assays against human cancer cell lines U251 were studied.

## 2. Results and Discussion

**Figure 1:**
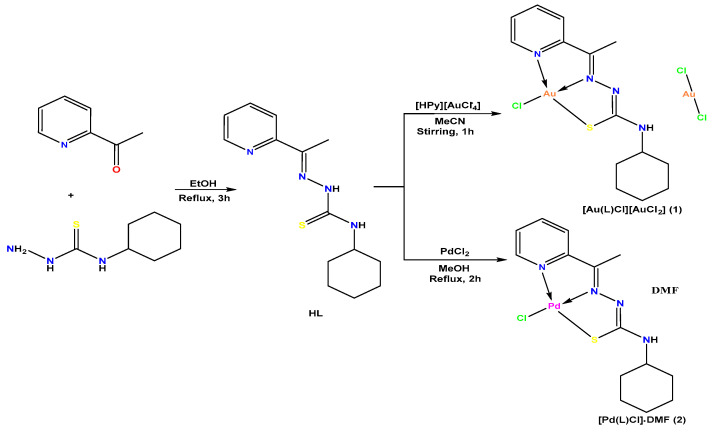
Synthesis of the thiosemicarbazone ligand HL and their complexes **(1)** and **(2)**.

The condensation reaction between N(4)-cyclohexyl-thiosemicarbazone with 2-acetylpyridine produced the tridentate Schiff base (HL). The complexation reactions of Au(III) or Pd(II) salts with HL yielded two novel metal complexes **(1)** and **(2)**, as shown in Figure 1. The complexes were characterized by X-ray diffraction analysis, spectroscopic and physicochemical analysis.

### 2.1. Spectroscopy Analysis

The comparison between the FTIR spectra of complexes **(1)** and **(2)** and the free ligand was important to evaluate the coordination trend (Figures S1–S3 in SI). A band of ν(C=N) of azomethine metal–nitrogen coordination of **(1)** and **(2)** is observed at 1528 and 1552 cm^−1^, respectively. Both complexes showed the folding characteristic of pyridine with the shift to lower wavenumbers. The ν(N-N) band shifted towards higher wavenumbers concerning those observed for HL resulting from the deprotonation of N(3)-H(3A) in both complexes and the increasing double bond character of the C-N bond acquired with the formation of the thiol tautomer with the coordination of the thiosemicarbazone to the metal centers.

The presence of the DMF molecule in **(2)** enabled the identification ν(C=O) band type at 1660 cm^−1^. Two bands were characterized by the presence of cyclohexyl in all compounds. In HL, these bands are identified at 2919 cm^−1^ and 2850 cm^−1^, while in **(1)** at 2928 cm^−1^ and 2845 cm^−1^ and in **(2)** at 2931 cm^−1^ and 2854 cm^−1^. The presence of these bands is compared with related works [26]. The values assigned to the bands in HL agree with what was observed in the literature [27,28]. The main bands of the FTIR spectra of complexes **(1)** and **(2)** and the free ligand are reported in Table 1.

Absorption spectroscopy in the UV–Vis region used a concentration of 2 × 10^−5^ mol/L in DMF for HL and their respective complexes **(1)** and **(2)**. In HL, a large band is observed at λ = 318 nm that may be associated with n → π* transitions and π → π*. This band is very close to the one presented by Demertzi et al. [29] in the study of the same Schiff base in CHCl_3_ with the same characteristic band at λ = 330 nm. In the coordination of thiosemicarbazone to the metal centers, a shift of the band transitions n → π* and π → π* for λ = 294 and 295 nm is observed in **(1)** and **(2)**, respectively.

Spectroscopic characterization using the ^1^H NMR of the studied compounds allowed us to compare the presence of protons in the structures and their behavior in solution (Figures S4-S6 in SI). The compounds HL and **(1)** were solubilized in CDCl_3_, while complex **(2)** was solubilized in DMSO-d_6_ due to their better solubility in these deuterated solvents. The presence of multiplets in opposite displacements referring to the terminal rings in thiosemicarbazone is observed in HL [30,31,32]. The cyclohexyl-thiosemicarbazone molecule is reported with their respective protons represented as two close multiplets that can be identified in their equatorial and axial form. The atoms of equatorial hydrogen would be at a chemical shift of (δH) 1.91 ppm, and the hydrogen atoms would be at a chemical shift of (δH) 1.31 ppm. In HL, equatorial hydrogen atoms are observed at (δH) 1.83 ppm, and the hydrogen atoms of axial hydrogens are observed at (δH) 1.43 ppm. The peaks of the protons present in the pyridine molecule are observed in a more unshielded spectrum region due to the presence of the nitrogen atom in the ring. Due to the number of hydrogen atoms in the very proximity and their coupling to each other, the peaks referring to these protons are observed in multiplets ranging from (δH) 7.90 to 7.40 ppm.

In the HL spectrum, the protons of the methyl group are observed in a more shielded chemical shift at (δH) 2.35 ppm in the form of a singlet with integration for the three hydrogen atoms. In complexes **(1)** and **(2)**, the cyclohexyl group continues to be observed in the same chemical region as HL.

### 2.2. Structural Analysis

**Figure 2:**
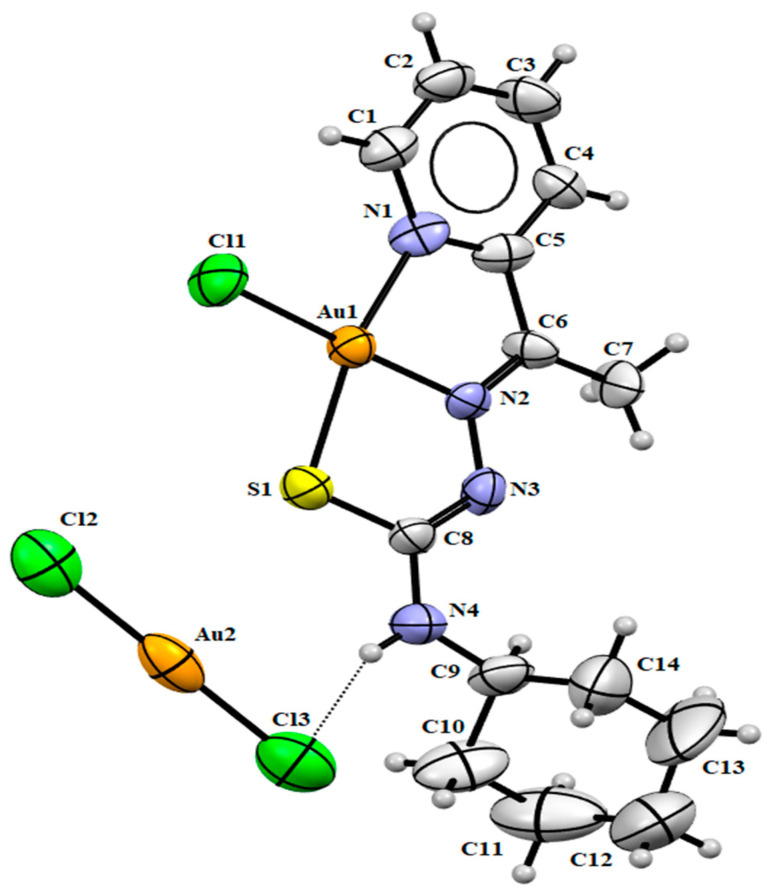
Molecular structure of **(1)** with crystallographic labeling (30% probability displacement). The intermolecular interaction is shown as a dashed line.

**Figure 3:**
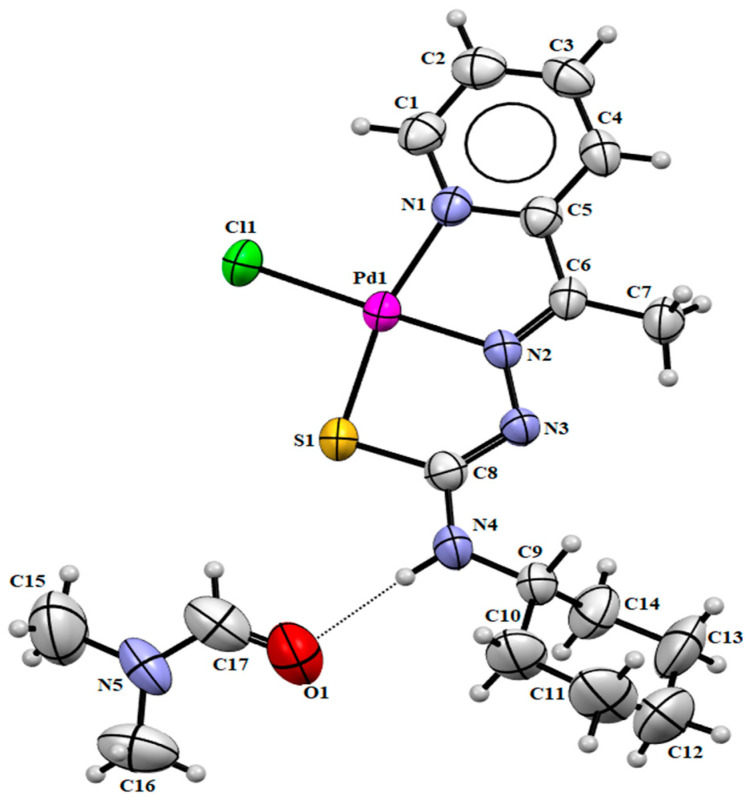
Molecular structure of **(2)** with crystallographic labeling (30% probability displacement). The intermolecular interaction is shown as a dashed line.

The structures of the newly synthesized gold(III) and palladium(II) complexes **(1)** and **(2)** were determined by the single crystal X-ray diffraction studies (Figure 2 and Figure 3). In complex **(1)**, the cationic part shows the gold(III) ion coordinated to the tridentate ligand through the nitrogen of the pyridine ring, the nitrogen of the azomethine group, the sulfur atom and the remaining binding site is occupied by the chloride ion forming a cationic complex. The asymmetric unit is completed with an anionic molecule [AuCl_2_]^−^ responsible for closing the load balance of the compound. In complex **(2)**, the palladium(II) atom is also coordinated by the *NNS*-donor atoms of mono-deprotonated thiosemicarbazone and has chloride as the fourth bonding atom. In the asymmetric unit, a DMF solvent molecule is still present. The Z conformation was identified concerning the C(6)-N(2) and C(8)-N(3) bonds in both complexes with the total conformation ZZ.

The trans angles N(1)-Au(1)-S(1) of 166.10(4)° and N(2)-Au(1)-Cl(1) of 178.40(5)° in structure **(1)** and the trans angles N(1)-Pd(1)-S(1) of 165.83(2)° and N(2)-Pd(1)-Cl(1) of 178.17(2)° in structure **(2)** deviate considerably from the ideal angle of 180°, distorting the square planar geometry of the complexes. The distorted square planar geometry in both complexes was calculated with the Okunievsky parameter [33], obtaining a value of 0.07 and consistent with the observed square planar geometry.

The thione tautomer observed in free ligand HL changes into thiol form when coordinated with the metal centers, concerning the observed experimental bond lengths of C(8)-S(1) and C(8)-N(3). The C(8)-S(1) bond length in HL of 1.679(2) Å, presenting a higher single bond character, increases to 1.770(2) Å in **(1)** and 1.755(6) Å in **(2)**. On the other hand, the bond length C(8)-N(3) is consistent with a double bond in HL, showing similar behavior in complexes **(1)** and **(2)** of 1.320(2) Å and 1.327(6) Å, respectively. In both complexes, the N(3) atom of the ligand molecule is deprotonated with the coordination. Therefore, the thiol tautomer formation is allowed, which is widely observed in other works that also present the deprotonation of thiosemicarbazone [25]. An electronic delocalization along thiosemicarbazone may also cause differences in the bond lengths.

The site position occupied by a chlorine atom shows a bond distance Au(1)-Cl(1) of 2.279(5) Å in the cationic part of **(1)** and a bond distance of Pd(1)-Cl(1) of 2.254(2) Å in **(2)**. However, the Au-Cl bond lengths of the anionic part of 2.244(8) and 2.259(8) Å are only marginally smaller. The bond distances and angles between gold or palladium with thiosemicarbazone are similar to the related compounds in the literature [34,35,36,37]. Selected bond lengths and bond angles are given in Table 2.

**Figure 4:**
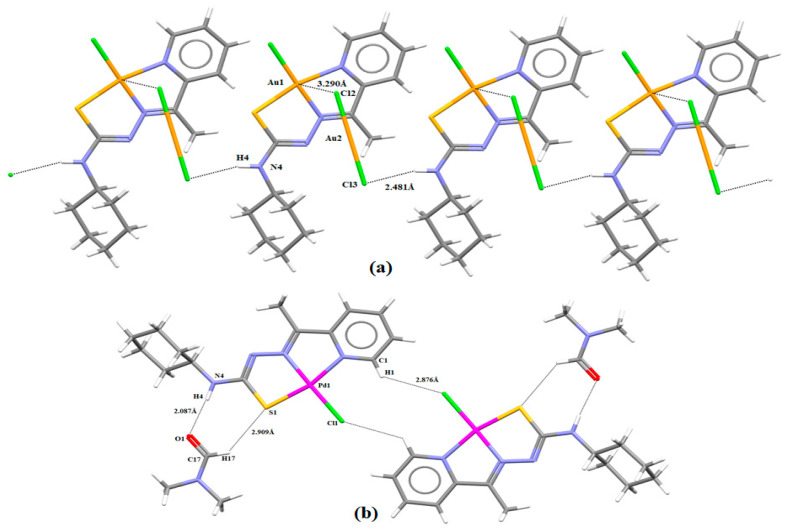
(**a**) Supramolecular structure generated by noncovalent interactions present in **(1)** in *b* axis; and (**b**) Intermolecular hydrogen interactions present in **(2)**. The hydrogen bonds are shown as a dashed line.

In **(1)**, the intermolecular hydrogen bond was observed between the N(4)-H(4A)∙∙∙Cl(3) atoms with a distance of 2.48 Å, [d(N∙∙∙Cl) = 3.298(2) Å, \N(4)-H(4A)∙∙∙Cl(3) = 159.1°; symmetry operation: x, 2 − y, 0.5 + z]. Weak interactions between Au(1)∙∙∙Cl(2) are observed with a distance of 3.290 Å. Additionally, cation–anion interactions and hydrogen bonds are observed (Figure 4a) and contribute to the formation of supramolecular architectures [38]. In **(2)**, the intermolecular hydrogens bonds were formed between the atoms N(4)-H(4)∙∙∙O(1) and C(17)-H(17)∙∙∙S(1) (Figure 4b). These interactions occur with the DMF molecule and the Pd(II) complex and are important for improving the stabilization of the crystal structure. Furthermore, a dimer is built by bifurcated intermolecular hydrogen bonds found between C-H···Cl hydrogen bonds between two molecules of the complex with the symmetry operator −x, 2 − y, 1 − z, as shown in Figure 4b.

### 2.3. Hirshfeld Surface

**Figure 5:**
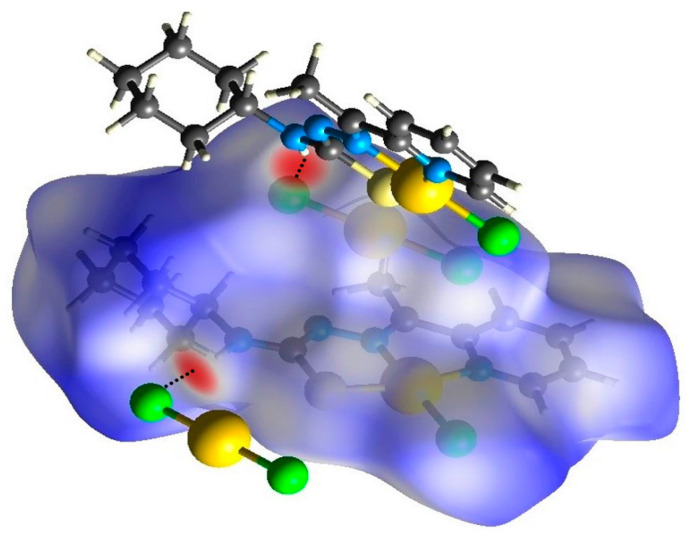
Hirshfeld surface of the complex **(1)** mapped with *d_norm_*.

**Figure 6:**
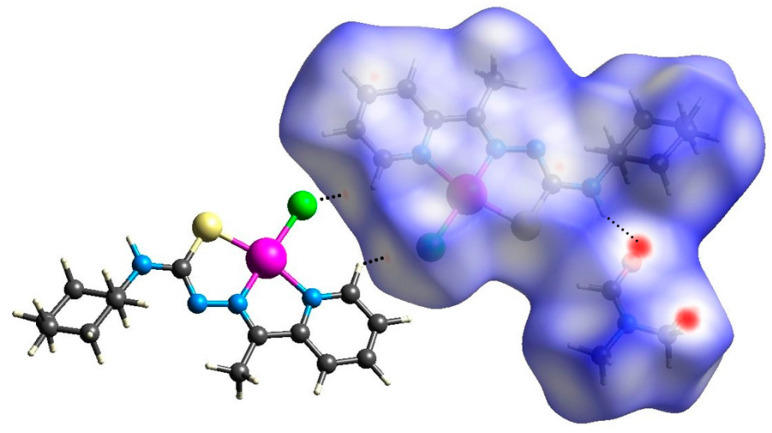
Hirshfeld surface of the complex **(2)** mapped with *d_norm_*.

**Figure 7:**
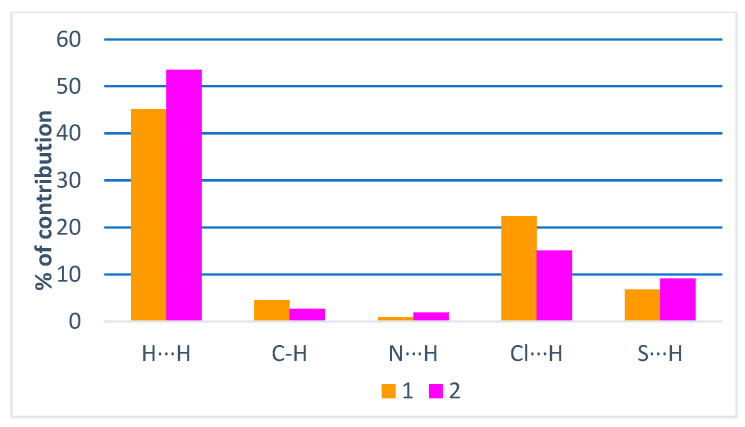
Percentage contribution of close contacts for complexes **(1)** and **(2)**.

The evaluation of the Hirshfeld surface [39,40] was performed using cif files generated from single-crystal X-ray diffraction with the intention of better understanding the formation of noncovalent interactions in **(1)** and **(2)**. According to the *d_norm_* map generated for **(1)** and **(2)**, both compounds present regions demarcated with red highlighted in the position in which they find the hydrogen atom of the terminal amine of thiosemicarbazone, characteristic of the previously observed, N(4)-(4A)∙∙∙Cl(3) and N(4)-H(4A)∙∙∙O(1), intermolecular hydrogen bonds (Figure 5 and Figure 6). An observation of this phenomenon occurring in the same positions, in both molecules, is a result of the similar form of coordination followed by the presence of stabilizing molecules: in the case of **(1)**, the anionic molecule [AuCl_2_]^−^; and in the case of **(2)**, the presence of dimethylformamide solvent. The shape index maps were generated but did not show distinct areas of π···π stacking connections or other strong interactions. The fingerprint maps for the main interactions were generated and showed their percentages that involve reciprocal contacts with a prevalence of H∙∙∙H and H∙∙∙Cl interactions. It is observed that H∙∙∙H interactions are more prevalent in **(2)** than in **(1)**. This observation results from the number of chloride ions present in **(1)**, which has the consequence of a higher prevalence of H∙∙∙Cl-type interactions concerning **(2)**. Despite the high electronegativity of the nitrogen atom, the interactions of the H∙∙∙N type were not as pronounced, given that nearly all of the nitrogen atoms along the molecules of **(1)** and **(2)** are in some way very involved in coordination. The interactions of the type H∙∙∙C are also observed in both structures. These interactions could be identified as non-classical hydrogen bonds that agree with those observed by the X-ray single-crystal diffraction. In addition, the contribution percentages referring to the fingerprint graphs are summarized in Figure 7, and the fingerprint plots of the quantitative data on the contacts (even distant ones) that most contribute to the formation of the crystals were obtained (Figures S7 and S8 in SI).

### 2.4. Biological Analysis

**Figure 8:**
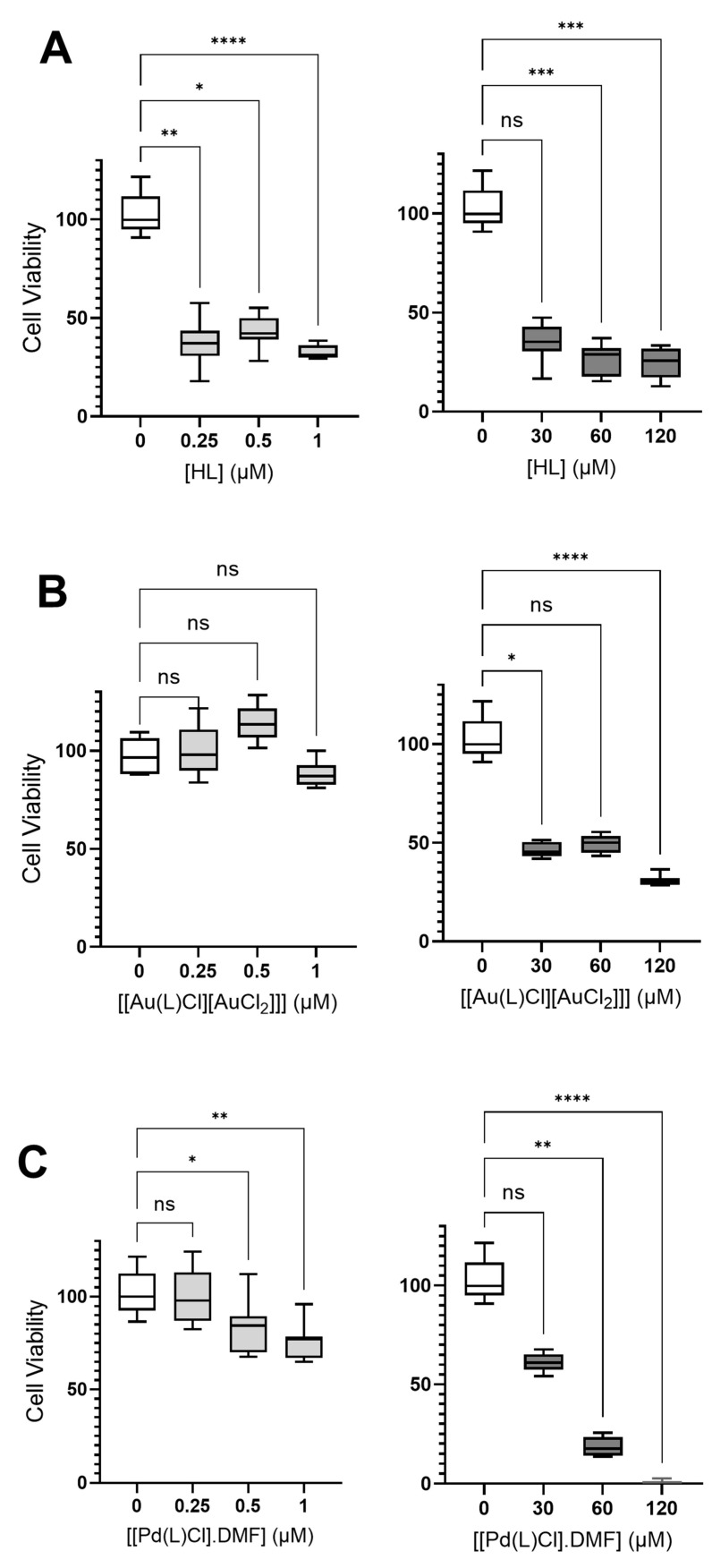
Evaluation of cytotoxic effects of 2-acetylpyridine N(4)-cyclohexyl-thiosemicarbazone ligand (HL) (**A**) and their two metal complexes, [Au(L)Cl][AuCl_2_] **(1)** (**B**) and [Pd(L)Cl]·DMF **(2)** (**C**) at low and high concentrations by MTT assay. DMSO at 0.01% was present in all groups, including the control. Graphs show a median with a quartile range, and asterisks indicate that the cell viability significantly differs from the respective control (* *p*-value < 0.05; ** *p*-value < 0.01; *** *p*-value < 0.005; **** *p*-value < 0.001 and ns = not significant).

The U251 human tumor cell line was used as an in vitro model for the screening of biological activity. The HL exhibited high toxicity at all concentrations analyzed, but not in a dose-dependent way. Moreover, even the highest concentration could not fully reduce the viability of tumor cells. Figure 8 shows the main results. These results indicate a nonspecific mechanism of toxicity.

**Figure 9:**
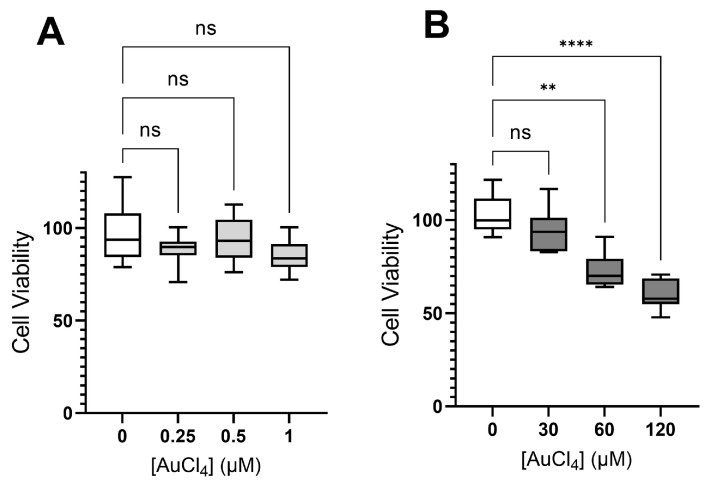
Evaluation of the cytotoxic effects of the compound AuCl_4_^−^ at low (**A**) and high (**B**) concentrations by MTT assay. DMSO at 0.01% was present in all groups, including the control. Graphs show a median with a quartile range, and asterisks indicate that the cell viability differs significantly from the respective control (** *p*-value < 0.01; **** *p*-value < 0.001 and ns = not significant).

Interestingly, both metal complexes **(1)** and **(2)** had a lower toxic effect than HL, especially at low concentrations. The compound [Hpy][AuCl_4_] presents moderate toxicity at very high concentrations (Figure 9). However, the complexation of the metal Au did not lead to the potentiation of the toxic effect of the thiosemicarbazone ligand (HL).

It was not possible to evaluate the toxic effect of PdCl_2_ because this compound was completely insoluble in the solvent used (and in other solvents compatible with cell cultures). Thus, the complexation of the thiosemicarbazone with the metal atom Pd gives bioavailability to palladium. In addition, and mainly, compound **(2)** shows a dose-dependent pattern in both low and high concentrations, reaching maximum efficiency at the highest tested concentration (Figure 8C). Many studies demonstrate the antitumor potential of Pd complexes, alone or associated with drugs [41,42,43,44].

### 2.5. Molecular Docking and ADME Analysis

To better understand the mechanism of interaction of the proposed compounds with the U251 cell, a docking study with two types of proteins that compose the U251 cell was performed. Docking functions are useful theoretical tools that can elucidate the intermolecular interactions between the residues of the active site of the protein and the small molecules.

The docking study reveals that the HL ligand is not a good inhibitor of PTPRZ and CRY2 enzymes. In both cases, it was observed that this molecule cannot interact properly with the important residues of the protein active sites. The score values for HL are almost half the value of the reference inhibitors (Table 3).

For the PTPRZ protein (PDB code 5AWX), the potent inhibitor SCB4308 has a profile of interactions with many hydrogen bond interactions, eight in total (Figure S9 in SI). All these hydrogen bonds are set with the residues of the enzyme active. Determining the ligand as a potent inhibitor requires considering certain properties of this tyrosine-phosphatase protein-like. Its interaction profile should repeat the same kind of interactions or at least interact with the same residues of its active site. The best-ranked molecule detected in the docking study was the inhibitor SCB4380, followed by the gold compound **(1)** and the palladium compound **(2)**. The difference in the binding energy values of compounds **(1)** and **(2)** is less than 2 kcal·mol^−1^, as shown in Table 3. This difference is mainly due to the two strong hydrogen bonds between the compound **(1)** and the Gln1977 residue.

**Figure 10:**
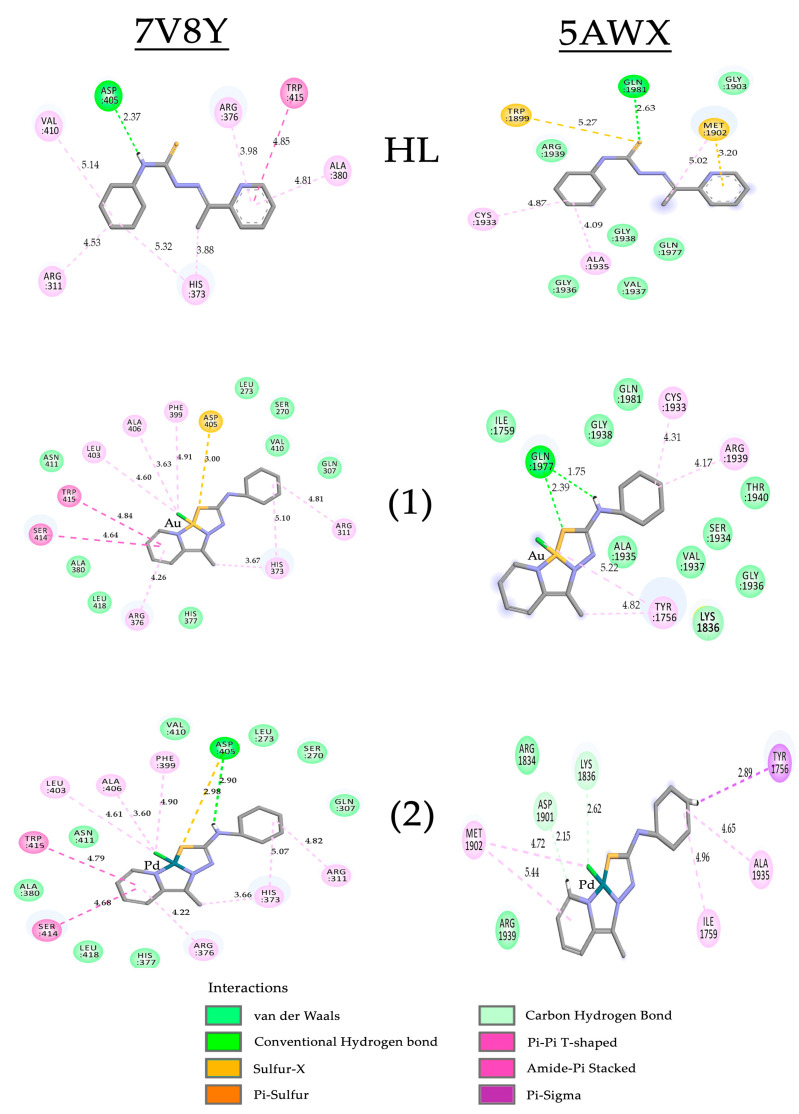
Two-dimensional representation of the main interactions and distances observed in the best-pose of docking result using the proposed protocol for the complex with the proteins CRY2 (PDB code 7V8Y) and PTPRZ (PDB code 5AWX) and the compounds HL, **(1)** and **(2)** (all distances are in Å).

The 2D interactions maps (Figure 10) indicate that for the CRY2 protein (7V8Y), the HL compound mainly makes the van der Walls interactions with the Arg376 (3.98 Å) and Trp417 (4.85 Å) residues. In the same set of residues, the inhibitor SHP1703 interacts with the residue Arg376 (3.47 Å) through a strong pi–cation interaction. The inhibitor also performs two hydrogen bond interactions (Figure S10 in the support information section) with the residues Ser414 (2.81 Å) and His377 (3.07 Å). For the CRY2 enzyme, compound **(2)** shows the energy of binding near the reference inhibitor (SHP1703), and the palladium compound shows a good interaction profile, as shown in Figure 10. The compound **(2)** can interact with all residues of the active site of the enzyme, imitating the same part of the SHP1703 ligand interactions. The gold compound **(1)** showed similar interactions as the compound **(2)**, except for the absence of a hydrogen bond with active site residues. It is well known that the ability to perform this type of interaction is important to increase the inhibition potential.

The ADME scores (Table S1 in SI), reveal that the HL ligand presents a huge difference in its ADME and bioactivity value scores compared with the reference inhibitors and the compounds **(1)** and **(2)**. The inability to cross the blood–brain barrier associated with the low value for the partition coefficient (logP) pushes the HL out of the set of molecules that can be indicated as a good inhibitor for glioblastomas cell.

In most ADME and bioactivity properties, compounds **(1)** and **(2)** showed good similarity with the inhibitors SHP1703. These two molecules were classified as ion channel modulators, able to cross the blood–brain barrier. This can explain the high binding energy for both molecules observed in the docking study of the CRY2 and PTPRZ enzymes.

## 3. Materials and Methods

### 3.1. Material, Methods and Instruments

Reagents and solvents employed were obtained from commercial sources and used as received (Merck, Brazil). The UV–Vis spectra were recorded using the Varian-Cary spectrophotometer (Agilent Technologies, CA, USA) with solutions produced in methanol with a concentration of 2 × 10^−5^ M. The Perkin Elmer/Series II 2400 (Perkin Elmer, Shelton, USA) analyzer was used to evaluate the elemental analysis of the studied substances. The infrared spectra were obtained with the aid of KBr (4000–400 cm^−1^) using the FTIR Varian 640 equipment (Agilent Technologies, CA, USA). The ESI-MS and ESI-MS/MS spectra were obtained by the AB Sciex TripleTOF 5600^+^ spectrometer in mode positive, 5500 V and 200 °C, with solutions at a concentration of 50 µM (methanol/dimethylformamide, ratio 99/1%) and 0.1% acetic acid (SCIEX, Framingham, USA). [HPy][AuCl_4_] was prepared according to the known literature [45].

### 3.2. Synthesis of 2-Acetylpyridine N(4)-Cyclohexyl-Thiosemicarbazone HL

The HL synthesis was prepared as described in similar previous works [46]. The N(4)-cyclohexyl-thiosemicarbazone, 346.56 mg (2 mmol), was dissolved in 30 mL of ethanol. Thereafter, 2-acetylpyridine 242.28 mg (2 mmol) was added. A system involving reflux and heating was maintained for approximately 2 h. Clear and colorless crystals were obtained. Yield: 67% (369 mg). Melting point: 144–145 °C. Elemental analysis calculated for C_14_H_20_N_4_S (%): C 60.84; H 7.29; N 20.27; found: C 61.22; H 7.25; N 19.83. Selected IR bands (KBr, ν/cm^−1^): ν(N-H) 3323 s, 3215 s; ν(C=S) 777; ν(C=N) 1564; ν(N-N) 1047 m; ν(Py) 656. λmax = 318 nm. ^1^H NMR (CDCl_3_) δ, ppm: 2.62 (s, 3H, C-CH_3_), 1.81–1.15 (m, 11H, N–C_6_H_11_), 7.90–7.40 (m, 4H, Py ring), 7.30 and 7.94 (s, 2H, N–H).

### 3.3. Synthesis of 2-Acetylpyridine N(4)-Dichloride Aurate(I) Cyclohexyl-Thiosemicarbazone) Gold Chloride(III), ***(1)***

The thiosemicarbazone HL (27,6 mg, 0.1 mmol) was dissolved in 5 mL of methanol and then added to a solution of [Hpy][AuCl_4_] (41.8 mg, 0.1 mmol) in acetonitrile (5 mL). The reaction mixture was stirred for 1 h at room temperature. Red crystals suitable for X-ray diffraction were obtained. Yield: 32% (25 mg). Melting point: 180–182 °C. Elemental analysis calculated for C_14_H_19_N_4_SCl_3_Au_2_ (%): C 21.68; H 2.47; N 7.91; found: C 21.73; H 2.61; N 8.17. Selected IR bands (KBr, ν/cm^−1^): ν(N-H) 3397 w; ν(C=S) 779 s; ν(C=N) 1528 s; ν(N-N) 1096 m; ν(Py) 647 m. λmax = 330 mm. ^1^H NMR (CDCl_3_) δ, ppm: 2.61 (s, 3H, C-CH_3_), 1.85–1.10 (m, 11H, N–C_6_H_11_), 7.98–7.40 (m, 4H, Py ring), 8.88 (m, H, N–H).

### 3.4. Synthesis of (2-Acetylpyridine N(4)-Cyclohexyl-Thiosemicarbazone) Palladium Chloride(II), ***(2)***

The compound PdCl_2_ (17.7 mg, 0.1 mmol) was dissolved in 5 mL methanol for 1 h under heat and reflux. HL (27,6 mg, 0.1 mmol) was dissolved in 5 mL of methanol and added to the previous solution. The mixture was heated under heat and reflux for 2 h. Yellow block-like crystals suitable for X-ray diffraction were obtained at room temperature after some days. Yield: 36% (19 mg). Melting point: 275–277 °C. Elemental analysis was calculated for C_14_H_19_ClN_4_SPd: C, 40.30; H, 4.59; N, 13.43; found: C, 40.39; H, 4.35; N, 13.45. Selected IR bands (KBr, ν/cm^−1^): ν(N-H) 3254 w; ν(C=S) 773 s; ν(C=N) 1552 s; ν(N-N) 1082 m; ν(Py) 620 m. λmax = 294 mm. ^1^H NMR (DMSO-d_6_) δ, ppm: 2.34 (s, 3H, C-CH_3_), 1.90–1.04 (m, 11H, N–C_6_H_11_), 8.18–7.53 (m, 4H, Py ring), 8.55 (s, H, N–H).

### 3.5. Crystal Structure Determinations

The crystallographic data were obtained with the Bruker CCD SMART APEX II (Bruker, Karlsruhe, Germany) single crystal diffractometer with Mo Kα radiation (0.71073 Å). SAINT was employed using SADABS [47] to scale the data and perform the multi-scan absorption correction. The Bruker Software Package SHELXTL (APEX3 v2017.3-03) [48] used the intrinsic phasing mode and, subsequently, the Fourier-difference map analysis yielded the positions of the non-hydrogen atoms. Further refinement of the structure was made possible through SHELXL-2016 [49]. Molecular graphics were generated via the OLEX2 software (v1.5) [50]. The refinement results, experimental details and crystal data are summarized in Table 4.

### 3.6. Computational Details

The Hirshfeld surface (HS) and 2D fingerprint graphics were generated with the CrystalExplorer 17.5 [51] program from the crystallographic data of crystalline structures (CIFs). The *d_norm_* (normalized contact distance) 3D surface is represented by a fixed core scale map ranging from −0.2206 (red) to 1.3448 (blue). There are three main parameters for mapping the Hirshfeld surface, using the *d_norm_* method, Equation (1): *d_i_* (contact distance between the inside of the atom and the closest point to the surface), *d_e_* (contact distance between the outside of the atom and the closest point to the surface) and the van der Walls radius of these contacts. The intermolecular interactions of the most varied types can be identified, quantified and expressed in percentages (by the amounts *d_i_* versus *d_e_*) using the calculations of 2D fingerprint plots within the program. The intermolecular type interactions observed in the structures and their respective distances were translated in the range of 0.4–3.0 Å. Interactions arising from contacts between rings were calculated using the S-shape index with a range of −1.0 to 1.0 according to Equation (2).

(1) $$dnorm=\frac{di-{ri}^{vdW}}{{ri}^{vdW}}+\frac{de-{ri}^{vdW}}{{ri}^{vdW}}$$

(2) $$S=\frac{-2}{\pi }\;{tan}^{-1}\frac{K1-K2}{K1+K2}$$

### 3.7. Biological Activity

#### 3.7.1. Cell Culture

The cytotoxic activity of the compounds was tested against the human glioma cell line U251 as an in vitro model for screening potential antitumor compounds. The cells were cultured in 10 cm diameter plastic plates under controlled conditions (a humid atmosphere of 5% CO_2_ at 37 °C) in DMEM culture medium supplemented with 10% (*v*/*v*) fetal bovine serum, penicillin (100 IU mL^−1^) and streptomycin (100 mg mL^−1^), which was replaced every two to three days.

#### 3.7.2. Cell Treatment and Analysis of Viability

Cell viability was measured by the 3-(4,5-dimethyl-2-thiazolyl)-2,5-diphenyl-2H-tetrazolium bromide (MTT) reduction method. This test is based on the ability of viable cells to metabolize yellow-colored through their mitochondrial dehydrogenases into the purple-stained formazan product. The cells were plated at a density of 10,000 cells per well (96-well plates were used) and then exposed to the compounds at concentrations ranging from 0 to 75 µM (N = 8). After 96 h, the culture medium was exchanged for the MTT solution, and the plates were incubated for two hours. After that, the cells were lysed for spectrophotometric quantification at a wavelength of 595 nm. Dimethyl sulfoxide (DMSO) was used as a diluent for all compounds and it was present (0.5%) in all groups, including the control. The results are expressed as a percentage of the control viability.

#### 3.7.3. Data Analysis

Data were graphically expressed as median and submitted to the non-parametric Kruskal–Wallis test followed by Dunn’s multiple comparisons test. Probability values of *p* < 0.05 were accepted as an indication of a statistically significant difference (* = *p* < 0.05, ** = *p* < 0.01, * = *p* < 0.001).

### 3.8. Molecular Docking and ADME Studies

To better understand the mechanism of interaction of the proposed compounds with the U251 cell, a docking study was performed with two types of proteins that compose the U251 cell proteomic system. One of the chosen proteins studied was the mammalian cryptochrome isoform, CRY2, a core circadian clock regulator enzyme. The 3D coordinates of the crystallographic structure of the CRY2, with a resolution of 1.90 Å, are deposited under the code 7V8Y, in the Protein Data Bank (PDB). This protein is presented as forming a complex with the ligand 1-[(2R)-3-[3,6-bis(fluorenyl)carbazol-9-YL]-2-oxidanyl-propyl]imidazolinidin-2-one, a potent inhibitor named SHP1703, this inhibitor showed good performance in the reduction in the viability of glioblastoma cells [52].

The second protein used in the docking studies is a tyrosine phosphatase receptor type Z (PTPRZ), detected with high expression in cases of aggressive malignant gliomas [53]. The 3D crystallographic structure of this PTPRZ, with a resolution of 1.86 Å, is deposited in the PDB under code 5AWX. In the case of this protein, there is not a direct complex formed with an inhibitor. In the active site of the enzyme there is a halogen ion, Br^−^. This ion is located in the same position as the sulfur atom of the naphthalene disulfonate group of the inhibitor Trisoduim 3-hydroxy-4-[(4-sulfonato-1-naphthyl)diazenyl]- 2,7naphthalenedisulfonate (named SCB4380), a potent and selective inhibitor of this protein, see Figure S1 and the supporting information section. The SCB4380 inhibitor was studied by Fujikawa and coworkers through docking and molecular dynamics simulations, and the best pose and all residues that make the main interactions with the PTPRZ active site properly shown in their work.

The performed docking study used the GoldScore algorithm implemented in the protein–ligand docking software GOLD (2022.3 CSD) [54]. This algorithm was used to rank the conformation of the best pose with the best score (ΔG of binding energy) between the compounds HL, **(1)**, **(2)** and the inhibitors SHP1703, and SCB4380, and the enzymes CRY2 (with the inhibitor SHP1703) and PTPRZ (with the inhibitor SCB4380).

The docking protocol was performed in two steps, which consist of a pre-docking study using the complexed inhibitor to choose and evaluate the set of parameters that cover the best pose/score that fit the ligand superposition according to its original coordinates in the crystallographic structure and correlate this pose with the lowest root-mean-square deviation (RMSD) values for both coordinates (original and docked). The pre-docking protocol was taken using fix/fix enzyme-ligand rotatable bonds. The protein structure was previously prepared, using the GOLD standard protocols.

The GOLD protocol was used to add the hydrogen atoms considering the best-predicted protonation state for the histidines, glutamates and aspartates residues. The best dihedral proposed rotation angle to all asparagines and glutamines residues was applied. In the present docking study, all water molecules were eliminated in the crystallographic structure of the enzymes, since no interaction with water molecules was described in the inhibition process for both proteins studied. The coordinates of the structures of the complexed ligands were extracted for being analyzed in the superposition check to indicate the better-fitted pose with the structures obtained from the docking studies.

The active site region of the enzymes was delimited taking into account the center of the molecule (and the Br^−^ ion coordinates) in the complex of the original crystallographic structure considering all residues with 12 Å of distance around the region of the inhibitor. The population settings were programmed to consider 100,000 operations of evaluations. The population size was selected as 150, the number of islands was 5, and the size of the niche was equal to 2. The genetic algorithm was set with the punctuation of crossover selected fixed at 95, the tax of allele mutation was set at 95 and the mutation frequency was considered at 10.

As shown in Figure S10 in the supporting information section, the superposition of the inhibitors is in an outstanding low deviation value. The RMSD value was found under 0.29 Å, and this low value (a good RMSD for docking validation should be under 2.00 Å) gives a reliable rate to perform our docking studies using others compounds, as the prosed organometallics ligands, HL, **(1)** and **(2)**. Comparing the interactions performed for the SHP1703 inhibitor in the crystallographic structure and the interactions performed in the docked pose obtained in this study, it is possible to affirm that the protocol parameters can properly perform the docking study. After establishing and validating the docking protocol, the second part of the docking study was carried out. This step was where the complex between the proteins CRY2 and PTPRZ and the compounds HL, **(1)** and **(2)** were studied.

The characterization of the adsorption, distribution, metabolism and excretion of the new compounds was performed in a theoretical ADME study using the program Molinspiration (“Molinspiration Cheminformatics”, [55]) in e SwissADME [56]. The studied parameters were used to be compared with the parameters of the known inhibitors of the enzymes CRY2 (SHP1703) and PTPRZ (SCB4380). The structural properties such as the molecular weight, the number of aromatic heavy atoms (non-hydrogen atoms), the number of rotatable bonds, number of h-bond donors and acceptors were described. The coefficient of partition (log P) and the blood–brain barrier permeability were estimated to check the ability of the molecules to cross the cell membranes, as well as the number of violations of the Lipinsk rules, and the enzymatic abilities as described in Table S1.

## 4. Conclusions

The new Pd(II) and Au(III) complexes with thiosemicarbazone ligand were elucidated by single-crystal X-ray diffraction and agreed with the spectroscopic study. The metal center is observed with square planar geometry, presenting the thiosemicarbazone tridentate with the *NNS*-donor system. The anionic [AuCl_2_]^−^ and DMF solvent molecules are present in the asymmetric unit of **(1)** and **(2)**, respectively, and act in the formation of noncovalent interactions. The Hirshfeld surface allowed the evaluation of the topography of intermolecular interactions and quantitative data on the contacts that most contribute to the crystal lattice formation. The presence of the cyclohexyl at the ligand was responsible for most H···H interactions found in the 2D fingerprints studied. Hence, our study shows thiosemicarbazone as a useful prototype for the synthesis of new metal complexes with antitumor activity and reinforces the potential of Pd complexes for increasing cancer treatment.

## Figures and Tables

**Table 1:** Experimental vibrational modes (cm^−1^) observed in the infrared spectra of HL, **(1)** and **(2)**.

	ν(N-H)	ν(C=N)	ν(N-N)	ν(C=S)	ν(Py)
HL	3323, 3215	1564	1047	777	656
**(1)**	3397	1528	1096	779	647
**(2)**	3254	1552	1082	773	620

**Table 2:** Selected bond distances (Å) and bond angles (°) for **(1)** and **(2)**.

(1)		(2)	
S(1)-C(8)	1.770(2)	S(1)-C(8)	1.755(6)
C(6)-N(2)	1.310(2)	C(6)-N(2)	1.295(6)
C(8)-N(3)	1.320(2)	C(8)-N(3)	1.327(6)
C(8)-N(4)	1.320(2)	C(8)-N(4)	1.459(6)
N(2)-N(3)	1.370(2)	N(2)-N(3)	1.369(6)
Au(1)-S(1)	2.261(5)	Pd(1)-S(1)	2.254(2)
Au(1)-N(1)	2.047(2)	Pd(1)-N(1)	2.046(5)
Au(1)-N(2)	1.961(2)	Pd(1)-N(2)	1.960(4)
Au(1)-Cl(1)	2.279(5)	Pd(1)-Cl(1)	2.254(2)
Au(2)-Cl(2)	2.259(8)	---	---
Au(2)-Cl(3)	2.244(8)	---	---
S(1)-Au(1)-Cl(1)	95.70(2)	S(1)-Pd(1)-Cl(1)	96.55(6)
N(1)-Au(1)-Cl(1)	98.20(4)	N(1)-Pd(1)-Cl(1)	97.63(2)
N(1)-Au(1)-S(1)	166.10(4)	N(1)-Pd(1)-S(1)	165.83(2)
N(2)-Au(1)-Cl(1)	178.40(5)	N(2)-Pd(1)-Cl(1)	178.17(2)

**Table 3:** Docking score values (binding energy) of the best pose ranked compounds using the GoldScore function and the main nearest residues of the active sites of the PTPRZ (PDB code 5AWX) and CRY2 (PDB 7V8Y) enzymes (interactions distances evaluated until 6 Å).

PDB		HL	(1)	(2)	SCB4380
*5AWX*	Score (Kcal·mol^−1^)	−17.68	−23.69	−22.41	−31.26
	Active site residues interactions	Trp1899, Met1902, Cys1933, Ala1935, Arg1938, Gln1977	Tyr1756, Lys1836, Cys1933, Ser1934, Ala1935, Arg1939, Gln1977	Tyr1756, Arg1834, Lys1836, Asp1901, Met1902, Ala1935, Arg1939	Tyr1756, Asn1758, Lys1836, Trp1899, Cys1933, Ser1934, Ala1935, Arg1939, Gln1977
	Hydrogen bonds (receptor-ligand)	1	2	0	8
*7V8Y*		**HL**	**(1)**	**(2)**	**SHP1703**
	Score (Kcal·mol^−1^)	−20.98	−24.30	−32.12	−39.69
	Active site residues	His373, Arg376, Asp405, Trp415	His373, Arg376, His377, Asp405, Asn411, Ser414, Trp415	His373, Arg376, his377, Asp405, Asn411, Ser414, Trp415	Trp310, Phe314, Arg376, His377, Asn411, Ser414, Trp415, Trp417
	Hydrogen bonds (receptor-ligand)	1	0	1	2

**Table 4:** X-ray structure data collection and refinement parameters for compounds **(1)** and **(2)**.

	(1)	(2)
Empirical formula	C_14_H_19_C_l3_N_4_SAu_2_	C_17_H_26_ClN_5_OPdS
Formula weight	775.67	490.34
Crystal system	Monoclinic	Triclinic
Space group	Pc	$P\overset{-}{1}$
a (Å)	12.667(16)	6.921(6)
b (Å)	7.468(9)	9.481(6)
c (Å)	11.789(15)	17.225(2)
α (°)	90	86.657(2)
β (°)	107.92(2)	79.740(2)
γ(°)	90	71.362(2)
V (Å^3^)	1061.3(2)	1053.6(2)
Z	2	2
ρ (Mg·cm^−3^)	2.427	1.546
μ (mm^−1^)	14.288	1.122
Reflections collected	22,894	23,892
Reflections unique/Rint	3737/0.106	3723/0.073
Data/restraints/param.	3737/2/219	2723/0/239
Absorption correction	multi-scan	multi-scan
Max/min transmission	0.977/0.866	0.288/0.065
Final R indices [I > 2σ(I)]	0.038/0.074	0.048/0.101
GooF	1.025	1.051
Largest diff. peak and hole (eÅ^−3^)	0.81/−0.81	0.53/−0.70

## Data Availability

The datasets generated for this study can be found in the Supplementary Material.

## Supplementary Materials

The following supporting information can be downloaded at: https://www.mdpi.com/article/10.3390/ijms241411442/s1. Crystallographic information files are available from the Cambridge Crystallographic Data Center upon request (http://www.ccdc.cam.ac.uk/structures, accessed on 9 July 2023, CCDC 2262708-2262709).
