# Supplementary material for: Crystal Design, Antitumor Activity and Molecular Docking of Novel Palladium(II) and Gold(III) Complexes with a Thiosemicarbazone Ligand
Source: Int J Mol Sci. 2023 Jul 14;24(14):11442. doi: 10.3390/ijms241411442 (PMC10380234; doi:10.3390/ijms241411442)
Supplement: Supplementary file 1 [file ijms-24-11442-s001.zip › ijms-2489354-supplementary.pdf]

Supplementary Material

Crystal Design, Antitumor Activity and Molecular Docking of Novel Palladium(II) and Gold(III) Complexes with a Thiosemicarbazone Ligand

Claudia C. Gatto [1,*], Carolane M. Almeida [1], Érica C. M. Nascimento [2], João B. L. Martins [2], Tales H. A. da Mota [3] and Diêgo M. de Oliveira [3]

[1] Laboratory of Inorganic Synthesis and Crystallography, Institute of Chemistry, University of Brasilia, Brasília 70904-970, Brazil
[2] Laboratory of Computational Chemistry, Institute of Chemistry, University of Brasilia, Brasília 70904-970, Brazil
[3] Multidisciplinary Laboratory of Human Health, Faculty UnB Ceilândia, University of Brasilia, Brasília 72220-275, Brazil
* Correspondence: ccgatto@unb.br; Tel.: +55-61-31073872; Fax: +55-61-31073900

SUMMARY

Figure S1. IR spectra of compound **HL**. III

Figure S2. IR spectra of compound **(1)**.. III

Figure S3. IR spectra of compound **(2)**.. III

Figure S4. ^{1}H NMR spectra of compound **HL** ($CDCl_3$,, 600 MHz, RT). IV

Figure S5. ^{1}H NMR spectra of compound **(1)** ($CDCl_3$, 600 MHz, RT). IV

Figure S6. ^{1}H NMR spectra of compound **(2)** (DMSO-d_6, 600 MHz, RT). IV

Figure S7. The two-dimensional fingerprint plots for **(1)**.. V

Figure S8. The two-dimensional fingerprint plots for **(2)**.. V

Figure S9. (A) Representation of the active site of PTPRZ enzyme (centre at Cys1933 residue) with the superposition of the Br^- ion (sphere in dark red) and the sulphur atom of the naphthalene disulfonate group of the SCB4380 inhibitor. (B) 2D representation of the main interactions and distances observed in the best-pose of docking result using the proposed protocol for the complex PTPRZ and SCB4380 inhibitor (all distances are in Å)..VI

Figure S10. Pre-docking: (A) 2D representation of the main interactions and distances between the residues of the CRY2 enzyme and its inhibitor SHP1703 (data directly obtained from the crystallographic structure, PDB code 7V8Y). (B) 2D representation of the main interactions and distances observed in the best pose of docking result using the proposed protocol. (C) Superposition between the heavy atoms of the inhibitor SHP1703 (coloured in blue) in the original coordinates coming from PDB structure, and the best-pose ranked by binding energy in from docking study for the same molecule.......................................VII

Table S1. ADME properties of the compounds HL, (1), (2), and the known inhibitors of the studied enzymes..VIII

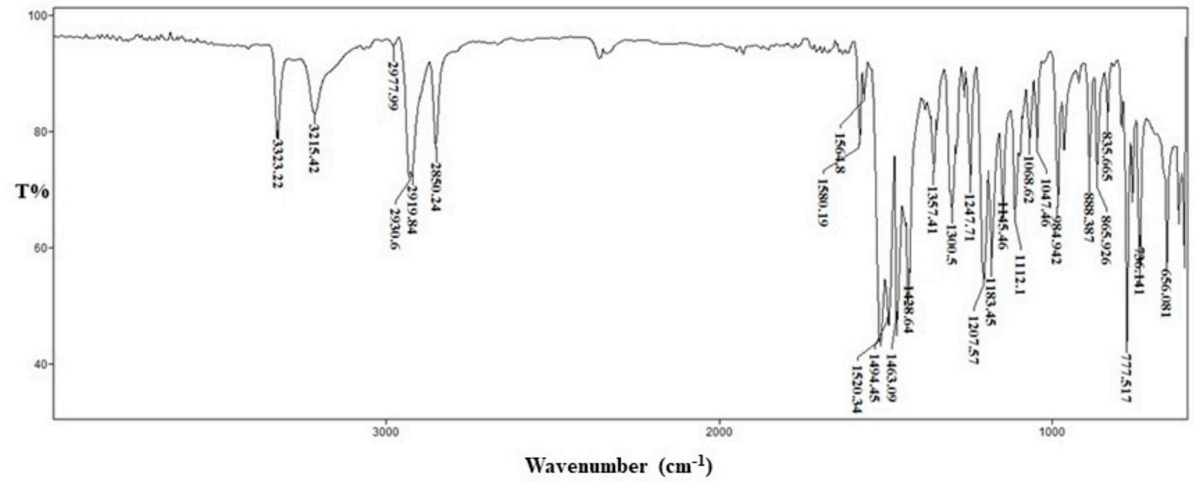

Figure S1. IR spectra of compound **HL**.

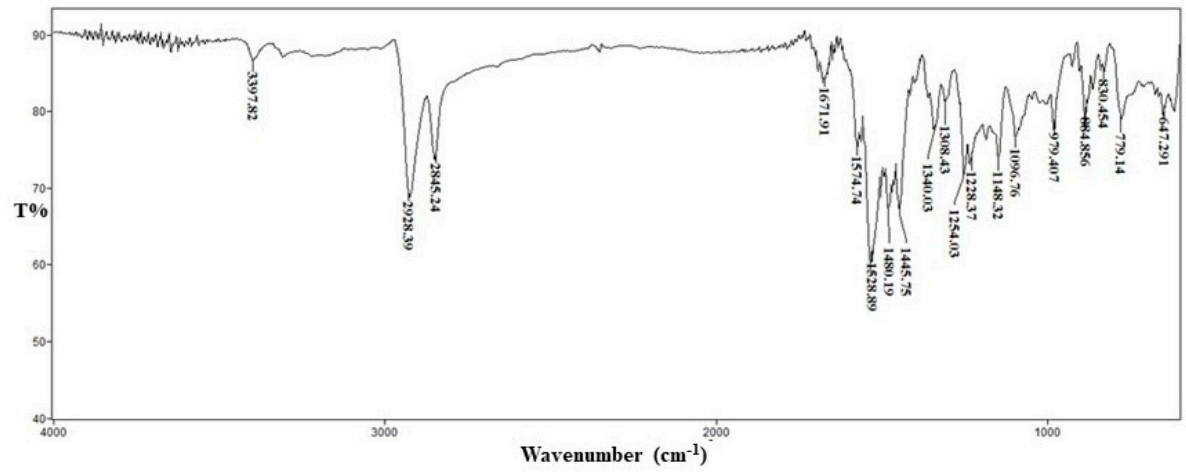

Figure S2. IR spectra of compound **(1)**.

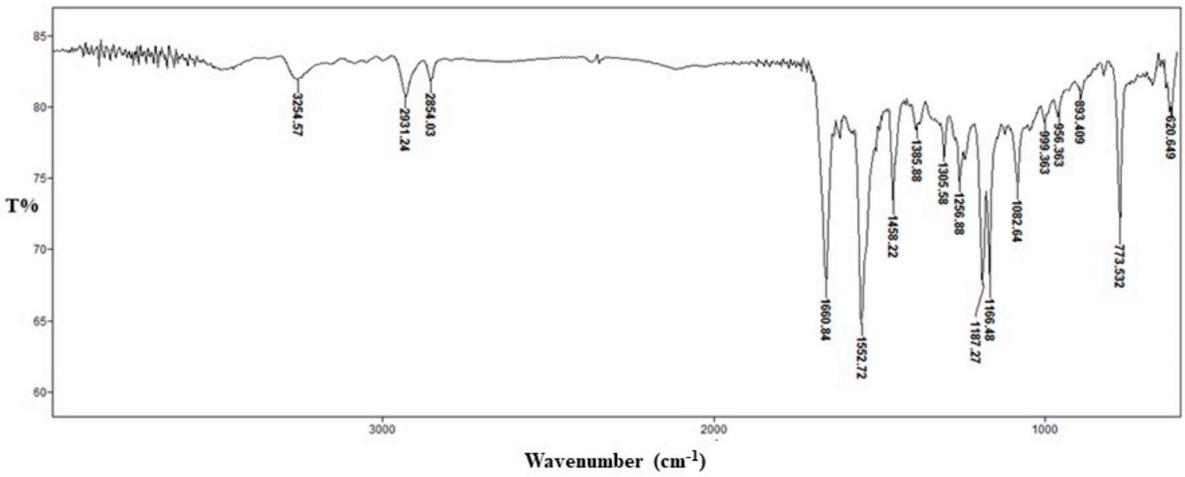

Figure S3. IR spectra of compound **(2)**.

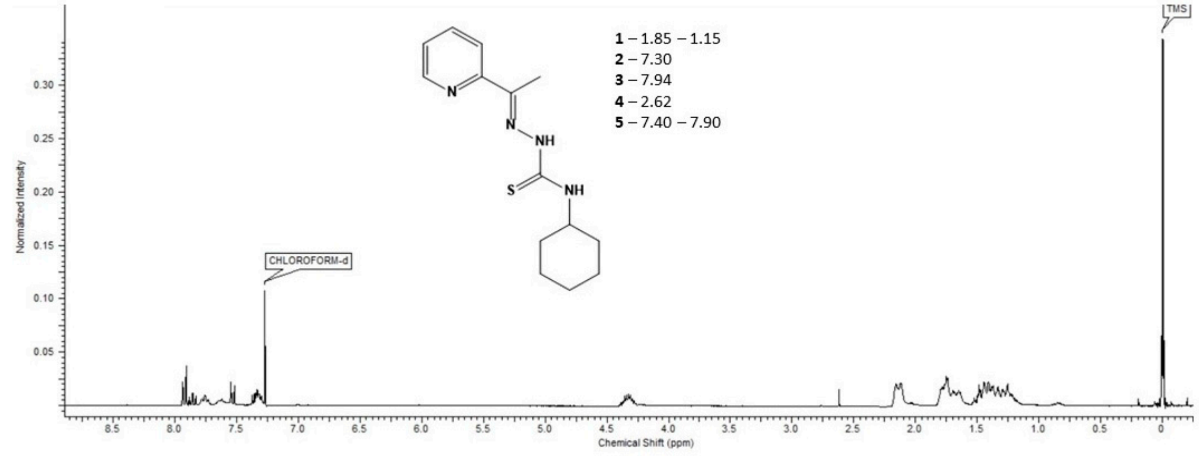

Figure S4. ^{1}H NMR spectra of compound **HL** ($CDCl_3$, 600 MHz, RT).

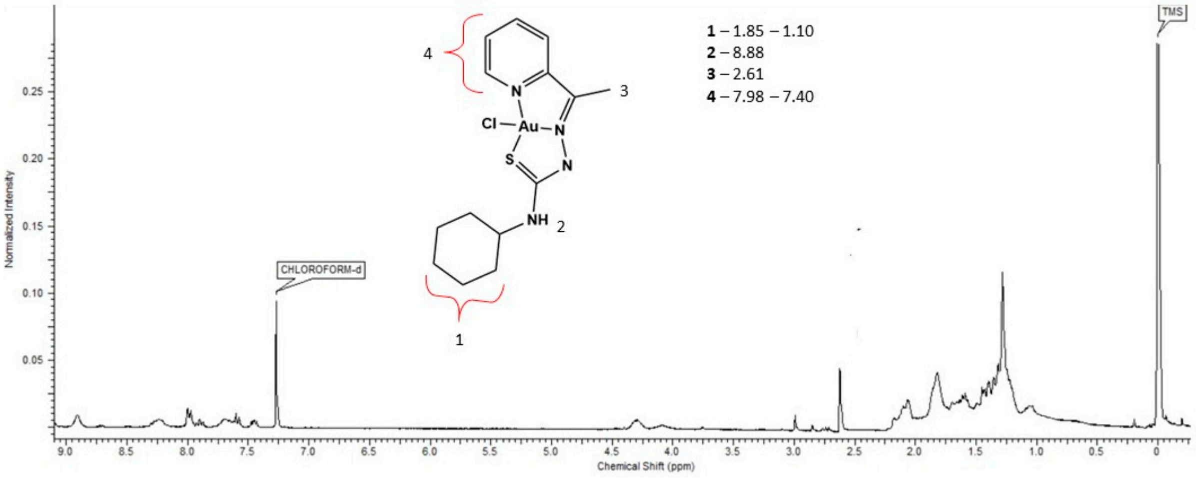

Figure S5. ^{1}H NMR spectra of compound **(1)** ($CDCl_3$, 600 MHz, RT).

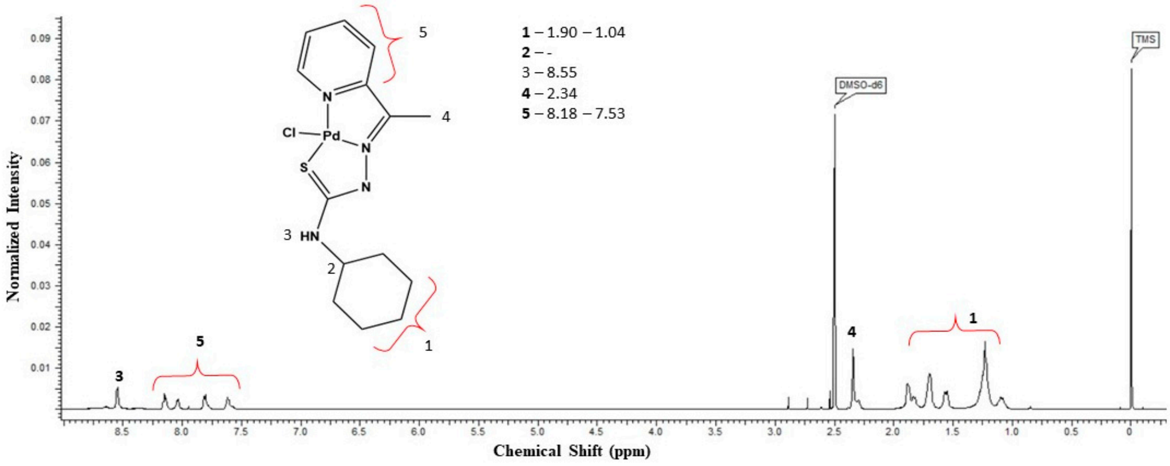

Figure S6. ^{1}H NMR spectra of compound **(2)** (DMSO-d_6, 600 MHz, RT).

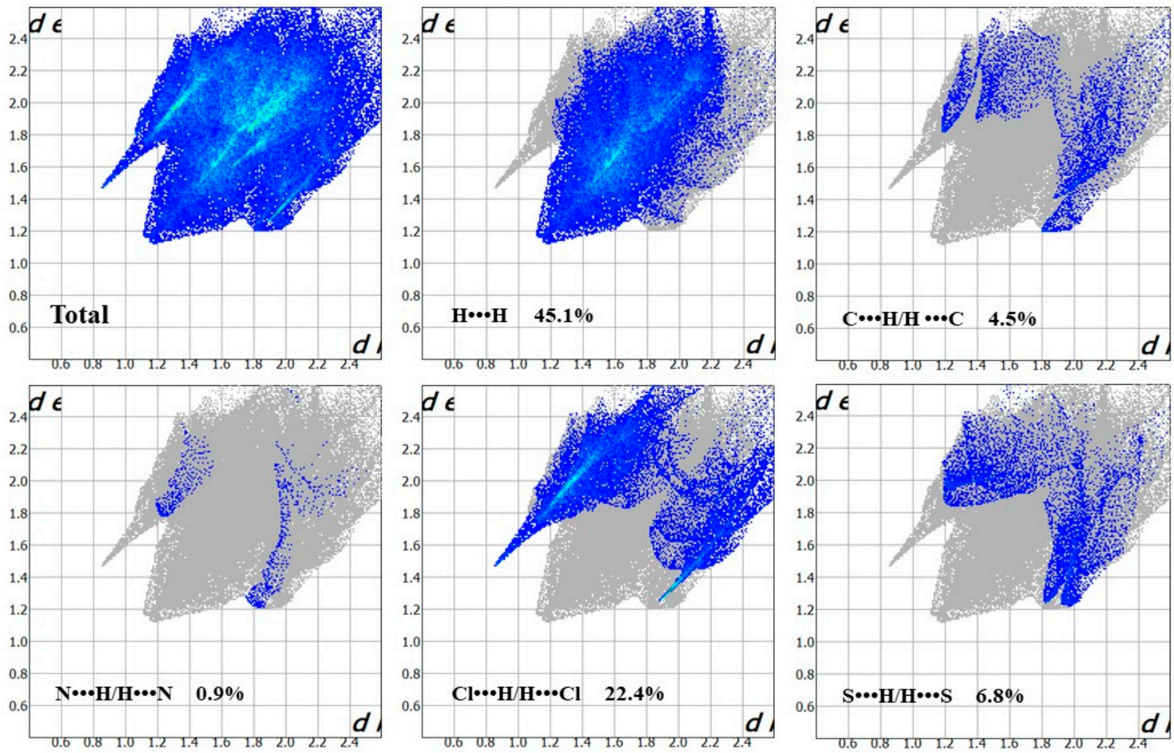

Figure S7. The two-dimensional fingerprint plots for **(1)**.

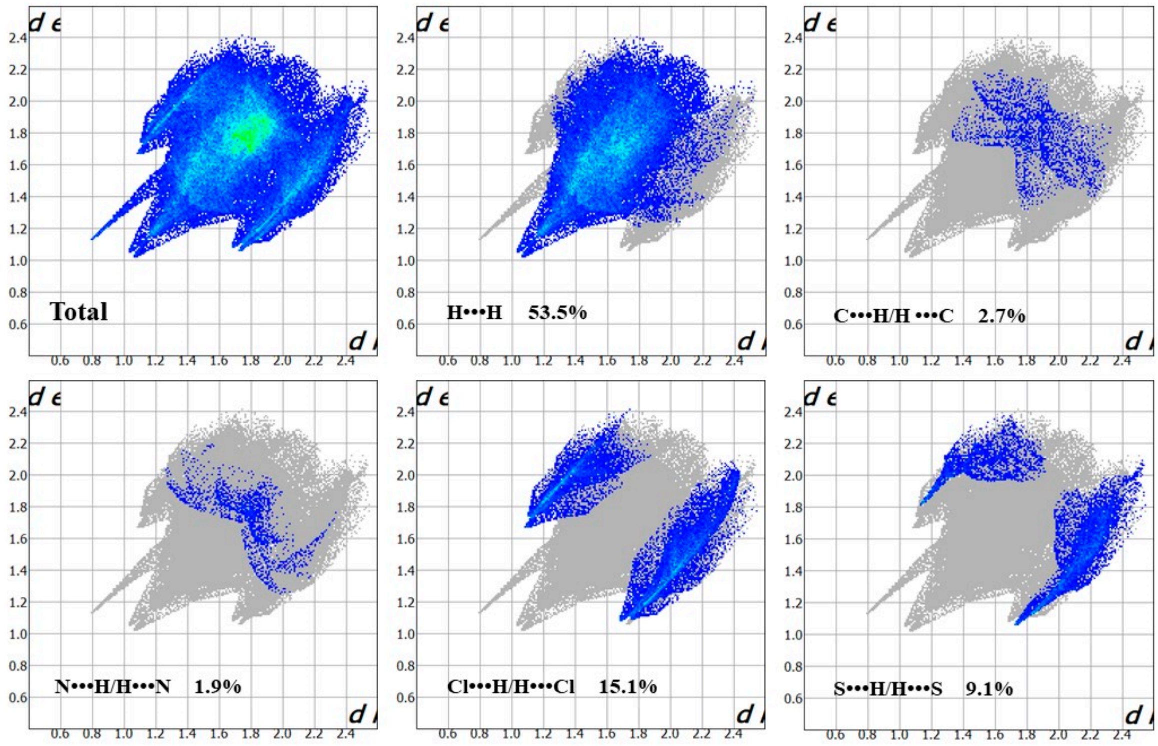

Figure S8. The two-dimensional fingerprint plots for **(2)**.

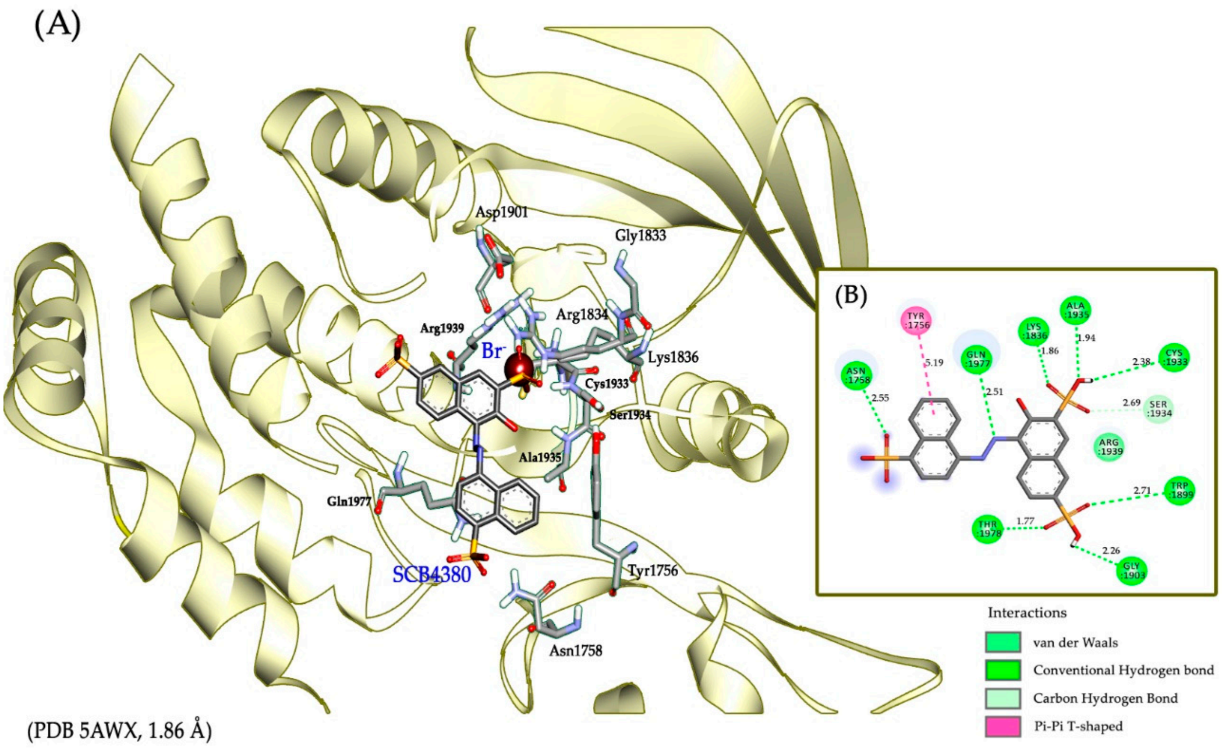

Figure S9. (A) Representation of the active site of PTPRZ enzyme (centre at Cys1933 residue) with the superposition of the Br^- ion (sphere in dark red) and the sulphur atom of the naphthalene disulfonate group of the SCB4380 inhibitor. (B) 2D representation of the main interactions and distances observed in the best-pose of docking result using the proposed protocol for the complex PTPRZ and SCB4380 inhibitor (all distances are in Å).

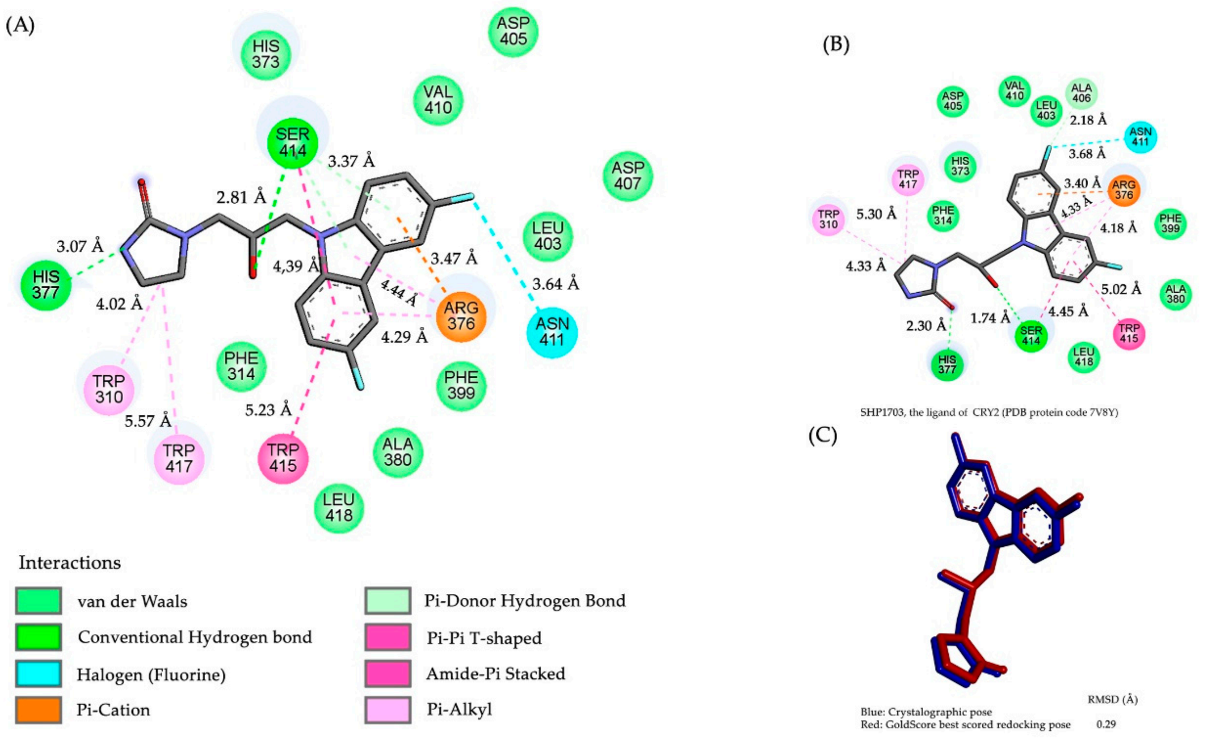

Figure S10. Pre-docking: (A) 2D representation of the main interactions and distances between the residues of the CRY2 enzyme and its inhibitor SHP1703 (data directly obtained from the crystallographic structure, PDB code 7V8Y). (B) 2D representation of the main interactions and distances observed in the best pose of docking result using the proposed protocol. (C) Superposition between the heavy atoms of the inhibitor SHP1703 (coloured in blue) in the original coordinates coming from PDB structure, and the best-pose ranked by binding energy in from docking study for the same molecule.

Table S1. ADME properties of the compounds HL, (1), (2), and the known inhibitors of the studied enzymes.

**ADME/Bioactivity score**	**HL**	**(1)**	**(2)**	**SCB4380**	**SHP1730**
GPCR ligand	-0.76	0.16	0.16	0.21	0.27
Ion channel modulator	-0.58	0.28	0.28	-0.01	0.02
Kinase inhibitor	-1.06	-0.43	-0.43	-0.11	0.14
Protease inhibitor	-0.58	-0.10	-0.21	0.18	0.02
Enzyme inhibitor	-0.12	0.16	0.16	0.15	-0.08
MW ($g.mol^{-1}$)	282.45	515.88	425.33	538.53	345.34
Aromatic heavy atoms	5	5	5	20	13
Rotatable bonds	5	2	2	5	4
H-bond acceptors	2	4	4	12	4
H-bond donors	3	2	2	4	2
logP	1.86	2.37	2.37	2.27	2.77
Lipinski violations	0	1	0	2	0
BBB permeant	No	Yes	Yes	No	Yes

The ADME scores shown in the Table S1, at the support information section, revels that the HL ligand presents huge difference in its ADME and bioactivity values scores comparing with the reference inhibitors and the compounds (1) and (2). The inability to cross the blood-brain barrier associated with the low value for the partition coefficient (logP) push the HL out of the set of molecules that can be indicate as a good inhibitors for glioblastomas cell.

The compounds (1) and (2), in the majority of ADME and bioactivity properties, shown good similarity with the inhibitors SHP1703. These two molecules were classified as Ion Chanel modulator, able to cross the blood-brain barrier; this can explain the high energy of binding for both molecules observed in the docking study of the CRY2 and PTPRZ enzyme.
